# Modification of Fatty Acid Composition of *Escherichia coli* by Co-Expression of Fatty Acid Desaturase and Thioesterase from *Arabidopsis thaliana*

**DOI:** 10.3390/bioengineering9120771

**Published:** 2022-12-06

**Authors:** Yihan Pu, Yujin Cao, Mo Xian

**Affiliations:** 1 The High School Affiliated to Renmin University of China, Beijing 100086, China; 2 CAS Key Laboratory of Biobased Materials, Qingdao Institute of Bioenergy and Bioprocess Technology, Chinese Academy of Sciences, Qingdao 266101, China

**Keywords:** AtFab2, AtFatA, fatty acid composition, unsaturated fatty acids, gas chromatography

## Abstract

Fatty acid composition has an important influence on the fluidity of biological membranes, which is a key factor for the survival of *Escherichia coli*. With the aim to modify fatty acid composition in this experimentally friendly microorganism, the *AtFab2* gene, encoding the *Arabidopsis thaliana* fatty acid desaturase, was expressed separately and jointly with AtFatA, a fatty acid thioesterase of the same plant origin. The expression of ATFab2 desaturase resulted in an enhancement of cis-vaccenic acid (18:1Δ11) contents, while amounts of palmitioleic acid (16:1Δ9) accumulated by *E. coli* were increased by 130% for the expression of the AtFatA thioesterase. In the final engineered strain co-expressing *AtFab2* and *AtFatA*, the percentage of palmitic acid (16:0), the most abundant saturated fatty acid found in *E. coli*, was reduced to 29.9% and the ratio of unsaturated fatty acid to saturated fatty acid reached 2:1. Free fatty acids accounted for about 40% of total fatty acid profiles in the recombinant strain expressing both two genes, and the unsaturated fatty acid contents reached nearly 75% in the free fatty acid profiles. The increase of unsaturated fatty acid level might provide some implication for the construction of cold tolerant strains.

## 1. Introduction

The fluidity of membrane lipids is necessary for cell growth and a variety of physiological functions. Unsaturated fatty acid (UFA) can increase the fluidity of the membrane because it is difficult to form a tight structure within the molecule. In general, the higher the degree of unsaturation, the greater the fluidity of membrane lipids [1]. Membrane fluidity is important for adapting to a low temperature environment. Due to the increased fluidity of the membrane, the membrane is still liquid at low temperature. Therefore, the cold resistance of the cell will be increased.

*Escherichia coli* has the type II fatty acid synthase (FAS II) system, which is present in most bacteria and plants [2]. This biosynthetic system is composed of a series of discrete and monofunctional enzymes. Fatty acids bind to acyl carrier protein (ACP) for the extension of the carbon chains in a sequential cycle [3]. However, the unsaturated fatty acid synthesis pathway in *E. coli* is different from higher plants. Wild-type *E. coli* does not contain any fatty acid desaturase. It uses an anaerobic pathway to synthesize UFAs. Fatty acid desaturases are enzymes that introduce double bonds into the hydrocarbon chains of fatty acids [4]. They are important for maintaining the proper structure and stability of biological membranes [5]. Plants have diverse fatty acid desaturases, many of which can use acyl-acyl carrier protein (ACP) as the substrates. The activities of these enzymes in prokaryotic hosts have been demonstrated by former researchers. For instance, expression of the *Hedera helix* desaturase in *E. coli* could synthesize C16 and C18 UFAs [6]. Cell extracts of *E. coli* expressing the acyl-ACP desaturase from *Macadamia integrifolia* showed enzymatic activity towards C16:0-ACP and C18:0-ACP [7]. The heterologous expression of the milkweed acyl-ACP desaturase led to Δ9 desaturation of the corresponding fatty acids [8]. Stearoyl-ACP desaturase (*AtFab2*) from *Arabidopsis thaliana* has previously been characterized in our laboratory [9]. The introduction of this fatty acid desaturase could significantly modify fatty acid composition of the engineered strain and increase the content of UFAs.

Acyl-ACP thioesterases can hydrolyze the thioester bond between the acyl moiety and ACP. These enzymes play an essential role in chain termination during de novo fatty acid synthesis in higher plants [10]. *E. coli* itself has two thioesterases, TesA and TesB, which belong to the acyl-coenzyme A (CoA) type thioesterase. They can cleave the thioester bond of fatty acyl-CoA and play an important role in fatty acid catabolism [11]. The catalytic activities of these two enzymes towards acyl-ACP esters are significantly lower than acyl-CoA esters [12]. On the other hand, there are a large number of acyl-ACP type thioesterases in plants. Many of them have been expressed and characterized in *E. coli*. The heterologous expression of a C8-10 specific thioesterase from *Cuphea hookeriana* in *E. coli* led to the accumulation of 84.9% C8 fatty acids [13]. The acyl-ACP thioesterase from *Macadamia tetraphylla* was expressed in *E. coli* and showed the highest activity towards 16:1-ACP [14]. The expression of plant acyl-ACP thioesterases in *E. coli* could lead to significant changes in the fatty acid composition [15,16,17]. *A. thaliana* has two isoforms of fatty acyl-ACP thioesterase, termed FatA and FatB. The substrate specificity of these isoforms depends on the chain length and saturation level of fatty acyl-ACPs. FatA prefers unsaturated oleoyl (18:1)-ACP as its optimal substrate while FatB has the highest activity towards palmitoyl (16:0)-ACP [18].

Based on this knowledge, we expressed the *AtFab2* and *AtFatA* (*A. thaliana* fatty acyl-ACP thioesterase) genes simultaneously in *E. coli* to investigate their co-effects on fatty acid composition. We expected unsaturated fatty acid contents in this recombinant strain could be enhanced by heterologous expression of these two enzymes associated with fatty acid biosynthesis.

## 2. Materials and Methods

### 2.1. Bacterial Strains, Plasmids, Culture Media and Conditions

A list of bacterial strains and recombinant plasmids used in this study is presented in Table 1. *A. thaliana*, Columbia ecotype, was used as the source for cloning the fatty acid desaturase and thioesterase genes. Chemically competent cells of *E. coli* DH5α and *E. coli* BL21(DE3) were purchased from TransGen Biotech (Beijing, China). The expression vectors of pET30a(+) and pACYCDuet-1 were obtained from Novagen (Madison, WI, USA). Liquid Luria-Bertani (LB) media or LB agar plates were used for DNA manipulation and protein expression. For fatty acid analysis, recombinant strains were cultured using M9 mineral medium (6 g/L Na_2_HPO_4_, 3 g/L KH_2_PO_4_, 1 g/L NH_4_Cl and 0.5 g/L NaCl) supplemented with 1 mM MgSO_4_ and 20 g/L glucose as the carbon source. 100 μg/mL of ampicillin or 50 μg/mL of kanamycin or 34 μg/mL chloramphenicol was added to the media if necessary.

### 2.2. Plasmids Construction

**Figure 1:**
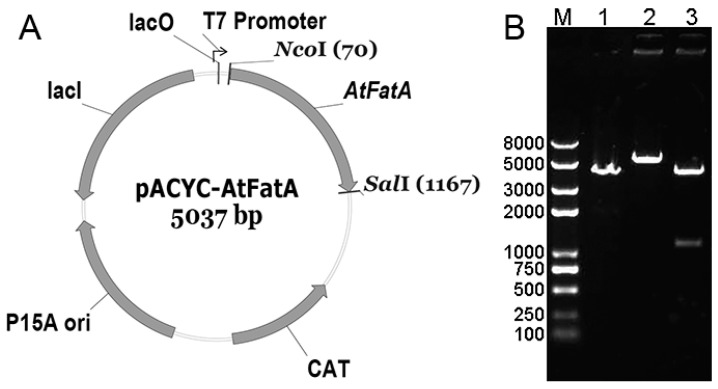
Construction of the recombinant plasmid expressing AtFatA. The AtFatA gene was inserted into the expression vector of pACYCDuet-1 between *Nco*I and *Sal*I. (**A**), Plasmid map of pACYCDuet-1; (**B**), Restriction map of recombinant pACYC-AtFatA, lane M: Trans2K Plus II DNA Marker; lane 1: pACYCDuet-1 digested with *Nco*I; lane 2: pACYC-AtFatA digested with *Nco*I; lane 2: pACYC-AtFatA digested with *Nco*I and *Sal*I.

The AtFab2 gene was cloned into vector pET30a(+) resulting recombinant plasmids pET-AtFab2 in our previous study [9]. Total RNA of *A. thaliana* was extracted from leaves using TRIzol reagent. The AtFatA (GenBank accession No. AK176105) gene was amplified using One Step RT-PCR (Takara, Dalian, China) with primer pairs AtFatA-F (CCATGGTTATGTTGAAGCTTTCGTGT) and AtFatA-R (GTCGACTTAACTTGAAGGCTTCTTTC) containing restriction sites of *Nco*I and *Sal*I. The amplified PCR products were directly ligated to the TA Cloning vector pUCm-T (Sangon, Shanghai, China), resulting pUCm-T-AtFatA. The ligation products were transformed into *E. coli* DH5a competent cells. Colonies grown on ampicillin plates were identified by colony PCR and subject to sequencing. The successfully constructed pUCm-T-AtFatA and pACYCduet-1 were digested with restriction enzymes *Nco*I and *Sal*I. The DNA fragments were separated by agarose gel electrophoresis and then recovered using a Gel Extraction Kit (Omega Bio-Tek, Norcross, GA, USA). The corresponding fragments were ligated by T4 DNA liagase, creating pACYC-AtFatA (Figure 1A).

### 2.3. Protein Expression and Gel Electrophoresis Analysis

The obtained plasmids pET-AtFab2 or pACYC-AtFatA or both were transformed into *E. coli* BL21(DE3) competent cells by heat shock method. The transformants were used to inoculate liquid LB broth containing appropriate antibiotics and grown overnight. The saturated culture was diluted at a ratio of 1:100 to fresh LB medium and incubated under the same conditions. When the absorbance at 600 nm (OD_600_) of the culture reached about 0.6, isopropylthiogalactoside (IPTG) was added to a final concentration of 0.1 mM to induce protein expression. The temperature of was switched to 30 °C and growth was continued for 3 h. The cells pelleted collected by centrifugation from 1 mL of culture were suspended in 100 μL of loading buffer, heated to 100 °C for 10 min and then analyzed by sodium dodecyl sulfate-polyacrylamide gel electrophoresis (SDS-PAGE).

### 2.4. Protein Purification

*E. coli* cells expressing the His-tagged fusion protein were harvested by centrifugation, washed with sterile double distilled water, and resuspended in 2 mL of binding buffer (20 mM Tris-HCl, 500 mM NaCl, 10% glycerol, 25 mM imidazole, pH 8.0). Then, the cells were disrupted by ultrasonication (130 W, 2 s/2 s) for 20 min. The mixture was centrifuged to remove cell debris and the supernatant collected was loaded onto a Ni2+-NTA affinity column (Invitrogen, Carlsbad, CA, USA). Nonspecific binding proteins were washed with binding buffer for three times. The His-tagged fusion ATFab2 protein was eluted with elution buffer (20 mM Tris-HCl, 500 mM NaCl, 10% glycerol, 200 mM imidazole, pH 8.0). The yields of purified His6-ATFab2 were then characterized by SDS-PAGE gels.

### 2.5. Molecular Modeling of the Enzymes

Homology molecular models of AtFab2 and AtFatA were made by AlphaFold2, one of the newest protein folding tools created based on artificial intelligence [19,20]. The web-server of AlphaFold2 (https://colab.research.google.com/github/sokrypton/ColabFold/blob/main/AlphaFold2.ipynb (accessed on 3 August 2022)) was used to predict tertiary structures of these two proteins without using close homologs. The fasta files with Uniprot sequence of AtFab2 and AtFatA were submitted to the AlphaFold2 server, respectively. The max_recycles parameter was set as 24 and other parameters were maintained as default. The results of structure models were manipulated using PyMOL Molecular Graphics System (version 2.6.0a0, Schrödinger, LLC) to create images.

### 2.6. Lipid Extraction and Thin Layer Chromatography Analysis

Lipids were extracted from pelleted cells following the procedure of Valeur et al. [21] with some modifications. Bacterial cell pellets harvested from 200 mL of fermentation broth were broken by vortex mixing with 5 mL of chloroform/methanol/water (2:1:0.8, by volume) for 3 min. The resulting mixture was left over night. The chloroform phase was regarded as the total lipid extract and it was evaporated to dryness under a stream of nitrogen.

Free fatty acids (FFAs) were separated from membrane phospholipids by thin layer chromatography (TLC) on glass plates coated with silica gel [22]. The developing solvents were composed of petroleum ether/diethyl ether/acetic acid (50:50:1, by volume). The bands of FFAs were identified by staining with iodine, recovered from the TLC plates, and resuspended in 2 mL of methanol/chloroform (2:1, by volume). The mixture was separated by centrifugation and the chloroform phase was collected as the FFAs proportion.

### 2.7. FAMEs Preparation and Gas Chromatography Analysis

Fatty acid methyl esters (FAMEs) were prepared using the method described by Lounds et al. [23]. Total lipids or FFAs extracted in the chloroform phase were evaporated with nitrogen, then suspended in 3 mL 3 M methanolic HCl and heated at 80 °C for 1 h in sealed tubes. Esterified fatty acids were extracted by addition of 1.5 mL of 0.9% (*w*/*v*) NaCl and 1 mL of n-hexane. FAMEs were analyzed by using gas chromatography (Varian 450-GC) equipped with a flame ionization detector (FID). An HP-5 column (30 m in length, internal diameter 0.32 mm, film thickness 0.25 μm) was used for separation and high-purity nitrogen was used as carrier gas with a flow rate of 1 mL/min. The column temperature program was composed of an initial hold at 100 °C for 5 min, ramping at 20 °C per min to 160 °C, followed by heating until 250 °C with 10 °C per min and a final hold at 250 °C for 3 min. The injector and FID detector temperatures were maintained at 250 °C and 300 °C, respectively. The injection volume was 1 μL and the split ratio was 1:10. The samples were injected into the column and FAME peaks were obtained.

## 3. Results

### 3.1. Expression of ATFab2 and AtFatA Proteins in E. coli

**Figure 2:**
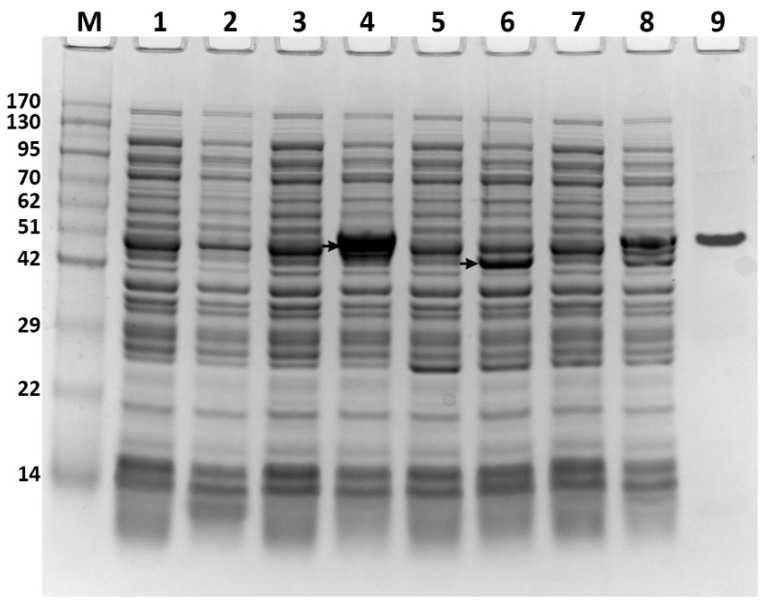
Analysis of the AtFab2 and AtFatA proteins expressed in *E. coli* by SDS-PAGE. Recombinant protein expression was induced by 0.1 mM IPTG for 3 h at 37 °C. Lane M, pre-stained protein molecular weight ladder; lane 1, uninduced cells extracts from *E. coli* BL21(DE3) harboring pET30a(+); lane 2, induced cells extracts from *E. coli* BL21(DE3) harboring pET30a(+); lane 3, uninduced cells extracts from *E. coli* BL21(DE3) harboring pET-AtFab2; lane 4, induced cells extracts from *E. coli* BL21(DE3) harboring pET-AtFab2; lane 5, uninduced cells extracts from *E. coli* BL21(DE3) harboring pACYC-AtFatA; lane 6, induced cells extracts from *E. coli* BL21(DE3) harboring pACYC-AtFatA; lane 7, uninduced cells extracts from *E. coli* BL21(DE3) harboring both pET-AtFab2 and pACYC-AtFatA; lane 8, induced cells extracts from *E. coli* BL21(DE3) harboring both pET-AtFab2 and pACYC-AtFatA; lane 9, purified recombinant His6-AtFab2. The positions corresponding to the AtFab2 and AtFatA proteins were indicated by arrows.

To express the ATFab2 and AtFatA proteins in *E. coli*, the coding regions of these two genes were cloned into the multiple cloning sites of pET30a(+) and pACYCduet-1 expression vectors, respectively. The expression constructs were verified by restriction enzyme digestion (Figure 1B) and direct DNA sequencing. The successfully constructed plasmids were then transformed into *E. coli* BL21(DE3) competent cells. The obtained recombinant strains were cultured in liquid LB media followed by induction with IPTG and the protein extracts were analyzed by SDS-PAGE. The recombinant proteins from different strains visualized by coomassie brilliant blue staining, shown in Figure 2. Protein bands of the expected size were detected when compared with the control strain *E. coli* BL21(DE3) harboring pET30a(+). After purification by nickel ion columns, the recombinant His6-ATFab2 protein revealed a single distinct band (Figure 2, lane 9, corresponding to the molecular weight of 50.5 kDa). In the ATFab2 and AtFatA co-expression strain, both two bands were detected.

**Figure 3:**
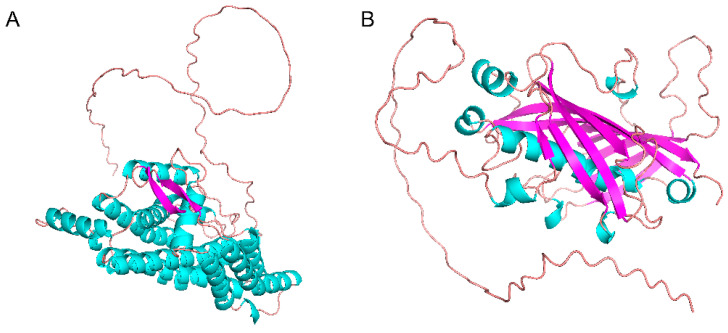
Predicted structures of the AtFab2 (**A**) and AtFatA (**B**) monomers by AlphaFold2. Both 3D structure models were predicted on AlphaFold2 server without using any homologs. Files of the predicted structures were shown as a ribbon diagram using PyMOL Molecular Graphic System.

We tested the feasibility of using AlphaFold2 to predict the structures of AtFab2 and AtFatA. Figure 3 showed the 3D structures of these two proteins. The predicted structure of ATFab2 was similar to that of the soluble plant stearoyl (18:0)-ACP desaturase from castor seed (PDB ID: 1AFR) [24]. When compared by the Protein Data Bank (PDB) Protein Structure Comparison Tool (https://www.rcsb.org/alignment (accessed on 4 August 2022)), the root-mean-square deviation (RMSD) value between them was 0.73, TM-score was 0.84 and sequence identity reached 89%. Meanwhile, the predicted structure of AtFatA was also like other acyl-ACP thioesterase. The RMSD between AtFatA and 12:0-ACP thioesterase from *Umbellularia californica* (PDB ID: 5X04) [25] was 3.2, TM-score was 0.68 and sequence identity was 40%, indicating that the structural similarity of these two thioesterases was less than that of the desaturases. Both AtFab2 and AtFatA should bind to their substrates, fatty acyl-ACPs to accomplish the catalyzed reactions. Therefore, their structures need to form fatty acid binding channels which play a critical role in their unique substrate specificity. The key residues in the substrate binding channel of AtFab2 showed high homology with previously identified Δ9 fatty acid desaturase [26]. Meanwhile, AtFatA should have similar substrate binding mode to other acyl-ACP thioesterases [27].

### 3.2. Effects of ATFab2 and AtFatA on Cell Growth

**Figure 4:**
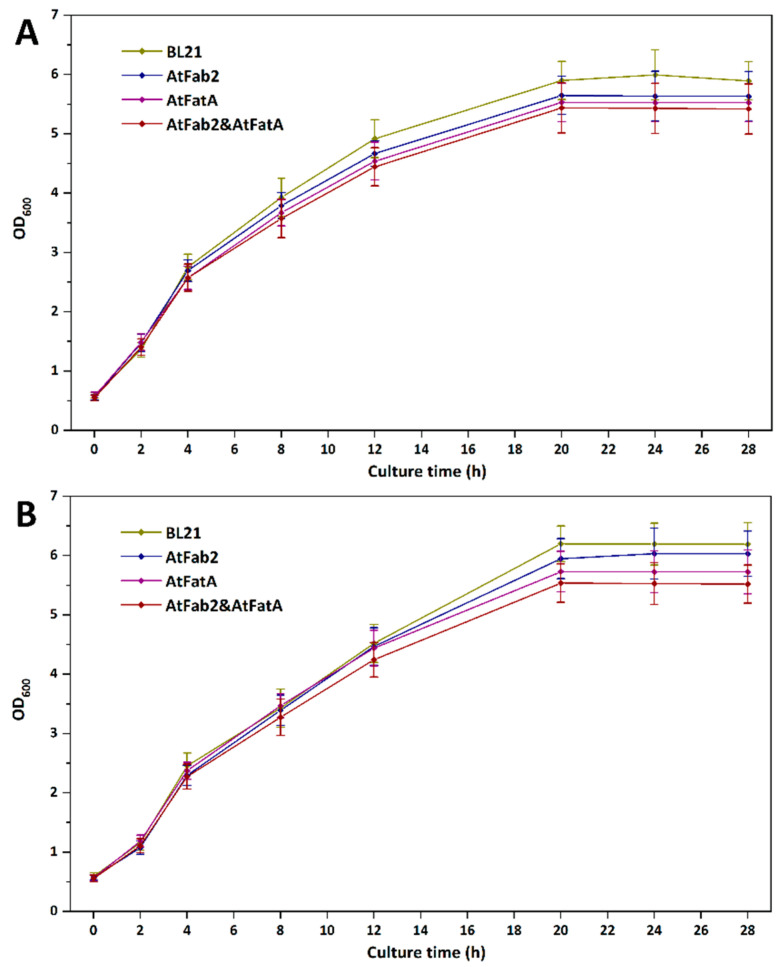
Effects of heterologous expression of AtFab2 and AtFatA on cell growth at different temperature. OD_600_ values were determined after being induced by IPTG. (**A**), growth curves of different strains at 37 °C; (**B**), growth curves of different strains at 30 °C. BL21, *E. coli* BL21(DE3) harboring pET30a(+); AtFab2, *E. coli* BL21(DE3) harboring pET-AtFab2; AtFatA, *E. coli* BL21(DE3) harboring pACYC-AtFatA; AtFab2&AtFatA, *E. coli* BL21(DE3) harboring both pET-AtFab2 and pACYC-AtFatA.

In order to study the effects of heterologous expression of ATFab2 and AtFatA enzymes on the growth of host cells, we cultured different strains under the same conditions. Figure 4 shows the growth curve of these strains at 37 °C and 30 °C. When these strains were cultured under the optimal temperature of 37 °C, they grew very fast. The OD_600_ of *E. coli* strains reached 6.0 or so in about 20 h and then maintained at this level. Compared with the wild-type strain BL21(DE3), the introduction of ATFab2 and AtFatA cannot promote cell growth. When the culture temperature was switched to 30 °C, all the strains grew slower than 37 °C, whereas the final cell densities were even higher than that of 37 °C. Cell densities of heterologous proteins overexpressed strains were also lower than strain BL21(DE3) harboring the empty vector pET30a(+). This might be due to that the introduction of foreign enzymes affected the growth of the strains and the increased levels of UFAs could not improve cell growth efficiency. Previous study showed that the fatty acid desaturase mutant strain of Bacillus subtilis revealed a cold-sensitive phenotype due to the dramatically increased SFA content [28]. However, the expression of the AtFab2 desaturase here only increased the UFA composition to some extent. Therefore, we cannot observe obvious cold tolerance of the engineered strain.

### 3.3. Changes of Fatty Acids Composition by Expressing ATFab2 and AtFatA

**Figure 5:**
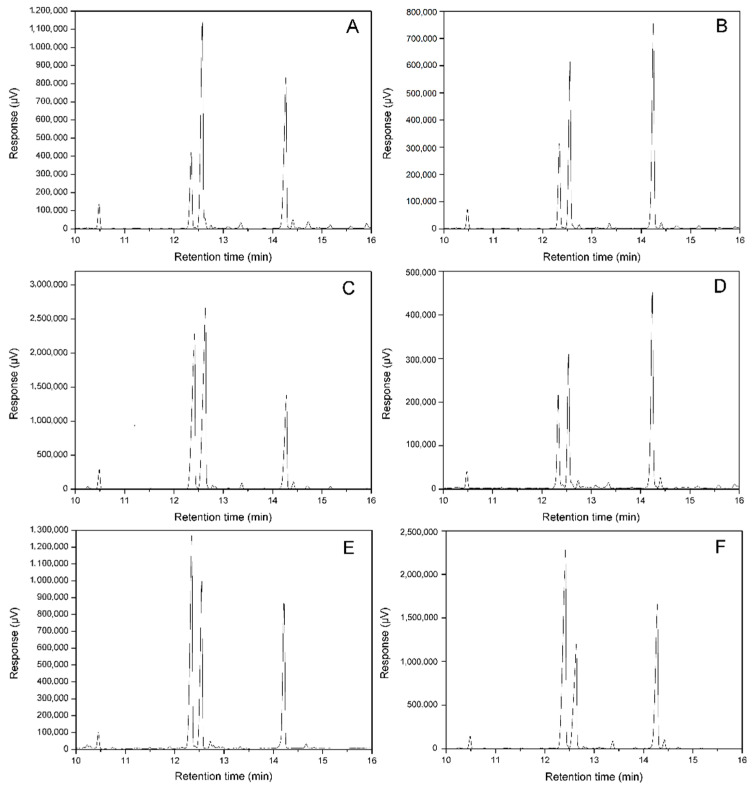
Gas chromatograms of fatty acid methyl esters obtained from different *E. coli* strains. (**A**), the control strain harboring pET30a(+); (**B**), the pET-AtFab2 transformants; (**C**), the pACYC-AtFatA transformants; (**D**), *E. coli* strain harboring both pET-AtFab2 and pACYC-AtFatA; (**E**), FFAs from the pET-AtFatA transformant; (**F**), FFAs from *E. coli* strain harboring both pET-AtFab2 and pACYC-AtFatA. The retention times of the fatty acid methyl esters were as follows: myristic acid, 10.48 min; palmitoleic acid, 12.33 min; palmitic acid, 12.55 min; cis-vaccenic acid, 14.24 min.

For the identification of fatty acids in *E. coli* by gas chromatography, mass spectrometry was performed in our previous study [29]. The fatty acid compositions of *E. coli* varied with the growth stages. Myristic acid, palmitic acid, palmitoleic acid and cis-vaccenic acid made up of the main fatty acid components in *E. coli* membrane lipids at the logarithmic phase. However, these two unsaturated fatty acids, palmitoleic acid and cis-vaccenic acid, were catalyzed by cyclopropane fatty acid synthetase to form methylene-9,10-hexadecanoic acid and methylene-11,12-octadecanoic acid during the transition from the logarithmic phase to the stationary phase. To avoid cyclopropane fatty acids formation, cultures of different recombinant *E. coli* strains were induced earlier at an OD_600_ of 0.3–0.4 and cells were harvested by centrifugation 3 h after induction (OD_600_ ≈ 1.0). Figure 5 shows the GC chromatograms of FAMEs from different recombinant strains and Table 2 shows the percentages of different fatty acids in different strains. The amount of each fatty acid was calculated according to the area of its peak. Our results indicated that different recombinant strains revealed different fatty acid compositions. Palmitic acid level of the control strain harboring the empty vector pET30a(+) reached 44.7 ± 1.0%, and the ratio of UFA (palmitoleic acid + cis-vaccenic acid) to saturated fatty acid (SFA, myristic acid + palmitic acid) was about 1:1 in this strain. For the recombinant strain carrying pET-AtFab2, palmitic acid content decreased to 34.4 ± 0.8% and the percentage of cis-vaccenic acid increased to 42.9 ± 0.8%. When the *AtFatA* gene was expressed separately, the recombinant strain overproduced palmitoleic acid. The level of this UFA was much higher (35.6 ± 0.6%) when compared with the control strain). For the strain co-expressing AtFab2 and AtFatA simultaneously, the percentage of palmitic acid further decreased to 29.6 ± 1.0%. Additionally, the ratio of UFA to SFA reached 2:1 in this recombinant strain.

### 3.4. Changes of Free Fatty Acids Composition by Expressing ATFab2 and AtFatA

To investigate the changes of FFAs composition by the introduction of AtFatA and ATFab2, we separated FFAs with membrane phospholipids by TLC. Then FFAs were derived and analyzed by gas chromatography. The control strain carrying pET30a(+) could hardly synthesize any FFAs (data not shown). For the recombinant strain heterogously expressing *AtFatA* gene, we detected significant FFAs production and palmitoleic acid made up 46.3 ± 0.9% of the total FFA profiles. When this thioesterase was co-expressed with the fatty acid desaturase ATFab2, the most abundant FFAs were palmitoleic acid and C18:1 fatty acid. These UFAs accounted for more than 75% of the total FFAs and the ratio of FFAs in total fatty acids reached about 40% in this recombinant strain. Moreover, myristoyl-ACP was not a preferential substrate for the thioesterase AtFatA and myristic acid accounted for less than 1% of the FFA profiles.

## 4. Discussion

Fatty acids perform a variety of important functions in bacterial cells, and fatty acid composition exerts a major influence on fluidity of biological membranes [30]. It is generally believed that the liquid-crystalline state of cellular membrane is essential for its regular function. Former studies have showed fatty acid composition of most bacteria was dependent on the growth conditions and responsible for the thermal resistance capability [31]. Low temperature can cause the irreversible integrity of biological membranes, from a fluid state to a rigid state. Cell vitality will be damaged under cold conditions [32]. Among all the fatty acid constitutes, unsaturated fatty acids possess the lowest melting points. They lower the transition temperature of the membrane when incorporated into the lipid bilayer, thereby maintaining biological membranes in a liquid expanded state and compensating for the decreased temperature [33]. Increasing UFA contents will enhance the low temperature adaptability of microorganisms, while extremely high SFA constitute seems to hamper the survival of most bacteria at low temperature. The increased level of UFAs in our engineered strain might provide some implication for constructing low-temperature resistant microorganisms.

Since both plants and bacteria contain the same type II fatty acid biosynthesis system, we introduced genes associated with fatty acid metabolism of plant origin into *E. coli* to investigate their effect on fatty acid composition. Plant stearoyl-ACP desaturase is the only soluble fatty acid desaturase which has been proved to function in bacterial systems [34]. The AtFab2 desaturase catalyzed the conversion of stearoyl-ACP to oleoyl-ACP and played an important role in the biosynthesis of UFAs in *A. thaliana* [35]. However, stearoyl-ACP, the best substrate for this enzyme, was not a major component in *E. coli* acyl-ACP pools. Therefore, AtFab2 catalyzed the desaturation of palmitoyl-ACP to generate palmitoleic acid. The overproduced palmitoleic acid was further elongated by β-ketoacyl-ACP synthase, resulting in an enhanced cis-vaccenic acid level. Furthermore, when the *A. thaliana* acyl-ACP thioesterase AtFatA was co-expressed with AtFab2 desaturase, the percentage of palmitoleic acid in the recombinant strain was greatly enhanced along with a slight decrease in palmitic acid content. *AtFatA* encodes thioesterases with a preference for unsaturated acyl-ACPs and with the highest activity towards 18:1Δ9-ACP. The catalytic efficiencies of AtFatA towards unsaturated fatty acyl-ACPs were greater than that towards saturated fatty acyl-ACPs [36]. Therefore, the ratio of palmitoleic acid in the recombinant strain expressing *AtFatA* was much higher than that of palmitic acid, although palmitoyl-ACP is more readily available in *E. coli*. On the other hand, oleic acid was not a major fatty acid composition of *E. coli* and cis-vaccenic acid seemed not to be a suitable substrate for AtFatA. Thus, expression of *AtFatA* individually even showed a decreased cis-vaccenic acid level.

*E. coli* cell membrane is composed of phospholipid bilayer in which fatty acid determines its fluidity and rheological properties. Many important functions, e.g., the maintenance of shape and structure, nutrients uptake and output, signal transduction and communication, are related to membrane fluidity [37]. The appropriate fluidity is necessary for *E. coli* to maintain cell morphology and normal metabolism. The ratio of UFAs in phospholipids is associated with membrane biophysical properties. The enrichment of UFAs content in the engineered strains can lead to changes in membrane fluidity, thus affecting the functions of membrane. The control of membrane fluidity by increasing UFA compositions in *E. coli* would enhance the tolerance to cold stress.

## 5. Conclusions

In conclusion, we have successfully expressed the fatty acid desaturase and thioesterase from *A. thaliana* in *E. coli* BL21(DE3). Since both two enzymes showed the ability to enhance unsaturated fatty acid contents, the recombinant strain harboring the two genes showed significant changes in fatty acid composition. Additionally, in this final engineered strain, palmitic acid contents were reduced to 29.9% and the ratio of UFA to SFA reached 2:1.

## Figures and Tables

**Table 1:** Strains and plasmids used in this study.

Strains or Plasmids	Genotype/Description	Sources
Strains		
*E. coli* DH5α	*huA2 lac(del)U169 phoA glnV44 Φ80’ lacZ(del)M15 gyrA96 recA1 relA1 endA1 thi-1 hsdR17*	TransGen
*E. coli* BL21(DE3)	*F^−^ ompT hsdS_B_* (r_B_^−^ m_B_^−^) *gal dcm rne131* (DE3)	TransGen
Plasmids		
pACYCDuet-1	*Cm^r^ oriP15A lacI^q^ T7p*	Novagen
pET30a(+)	*Kan^r^ oripBR322 lacI^q^ T7p*	Novagen
pUCm-T	*Amp^r^ oripUC lacZ*	Sangon
pET-AtFab2	pET30a(+) harboring *A. thaliana* fatty acid desaturase	[7]
pUCm-T-AtFatA	pUCm-T harboring *A. thaliana* thioesterase	This study
pACYC-AtFatA	pACYCDuet-1 harboring *A. thaliana* thioesterase	This study

**Table 2:** Fatty acid compositions of different *E. coli* strains.

Strains	Myristic Acid	Palmitoleic Acid	Palmitic Acid	cis-Vaccenic Acid
	Fatty Acids (%)			
BL21	4.1 ± 0.3	20.2 ± 0.9	44.7 ± 1.0	31.0 ± 1.0
BL21/AtFab2	3.1 ± 0.4	19.6 ± 0.9	34.4 ± 0.8	42.9 ± 0.8
BL21/AtFatA	2.9 ± 0.3	35.6 ± 0.6	38.7 ± 0.7	22.8 ± 1.4
BL21/AtFab2&AtFatA	3.0 ± 0.2	21.0 ± 1.0	29.6 ± 1.0	46.4 ± 1.7
	**Free fatty acids (%)**			
BL21/AtFatA	1.4 ± 0.2	46.3 ± 0.9	31.4 ± 0.6	20.9 ± 0.7
BL21/AtFab2&AtFatA	0.9 ± 0.1	47.1 ± 0.7	22.9 ± 0.5	29.1 ± 0.5

All experiments were performed in triplicate and data were shown as the mean of three independent experiments with standard deviation.

## Data Availability

No new data were created or analyzed in this study. Data sharing is not applicable to this article.

## References

[1] Lee J.I., Kim S.S., Kang D.H. (2019). Susceptibility of *Escherichia coli* O157:H7 grown at low temperatures to the krypton-chlorine excilamp. Sci. Rep..

[2] Magnuson K., Jackowski S., Rock C.O., Cronan J.E. (1993). Regulation of fatty acid biosynthesis in *Escherichia coli*. Microbiol. Rev..

[3] Marrakchi H., Zhang Y.M., Rock C.O. (2002). Mechanistic diversity and regulation of Type II fatty acid synthesis. Biochem. Soc. Trans..

[4] Halim N.F.A.A., Ali M.S.M., Leow A.T.C., Rahman R.N.Z.R.A. (2022). Membrane fatty acid desaturase: Biosynthesis, mechanism, and architecture. Appl. Microbiol. Biotechnol..

[5] Los D.A., Murata N. (1998). Structure and expression of fatty acid desaturases. Biochim. Biophys. Acta-Lipids Lipid Metab..

[6] Whittle E., Cahoon E.B., Subrahmanyam S., Shanklin J. (2005). A multifunctional acyl-acyl carrier protein desaturase from *Hedera helix* L. (English ivy) can synthesize 16-and 18-carbon monoene and diene products. J. Biol. Chem..

[7] Gummeson P.O., Lenman M., Lee M., Singh S., Stymne S. (2000). Characterisation of acyl-ACP desaturases from *Macadamia integrifolia* Maiden & Betche and *Nerium oleander* L.. Plant Sci..

[8] Cahoon E.B., Coughlan S.J., Shanklin J. (1997). Characterization of a structurally and functionally diverged acyl-acyl carrier protein desaturase from milkweed seed. Plant Mol. Biol..

[9] Cao Y., Xian M., Yang J., Xu X., Liu W., Li L. (2010). Heterologous expression of stearoyl-acyl carrier protein desaturase (S-ACP-DES) from *Arabidopsis thaliana* in *Escherichia coli*. Protein Expr. Purif..

[10] Jones A., Davies H.M., Voelker T.A. (1995). Palmitoyl-acyl carrier protein (ACP) thioesterase and the evolutionary origin of plant acyl-ACP thioesterases. Plant Cell.

[11] Cho H.S., Cronan J.E. (1993). *Escherichia coli* thioesterase, I, molecular cloning and sequencing of the structural gene and identification as a periplasmic enzyme. J. Biol. Chem..

[12] Spencer A.K., Greenspan A.D., Cronan J.E. (1978). Thioesterases I and II of *Escherichia coli*. J. Biol. Chem..

[13] Feng Y., Zhang Y., Wang Y., Liu J., Liu Y., Cao X., Xue S. (2018). Tuning of acyl-ACP thioesterase activity directed for tailored fatty acid synthesis. Appl. Microbiol. Biotechnol..

[14] Moreno-Pérez A.J., Sánchez-García A., Salas J.J., Garcés R., Martínez-Force E. (2011). Acyl-ACP thioesterases from macadamia (*Macadamia tetraphylla*) nuts: Cloning, characterization and their impact on oil composition. Plant Physiol. Biochem..

[15] Huynh T.T., Pirtle R.M., Chapman K.D. (2002). Expression of a *Gossypium hirsutum* cDNA encoding a FatB palmitoyl-acyl carrier protein thioesterase in *Escherichia coli*. Plant Physiol. Biochem..

[16] Jha J.K., Maiti M.K., Bhattacharjee A., Basu A., Sen P.C., Sen S.K. (2006). Cloning and functional expression of an acyl-ACP thioesterase FatB type from *Diploknema (Madhuca) butyracea* seeds in *Escherichia coli*. Plant Physiol. Biochem..

[17] Voelker T.A., Davies H.M. (1994). Alteration of the specificity and regulation of fatty-acid synthesis of *Escherichia coli* by expression of a plant medium-chain acyl-acyl carrier protein thioesterase. J. Bacteriol..

[18] Bonaventure G., Bao X., Ohlrogge J., Pollard M. (2004). Metabolic responses to the reduction in palmitate caused by disruption of the FATB gene in *Arabidopsis*. Plant Physiol..

[19] Jumper J., Evans R., Pritzel A., Green T., Figurnov M., Ronneberger O., Tunyasuvunakool K., Bates R., Žídek A., Potapenko A. (2021). Highly accurate protein structure prediction with AlphaFold. Nature.

[20] Mirdita M., Schütze K., Moriwaki Y., Heo L., Ovchinnikov S., Steinegger M. (2022). ColabFold: Making protein folding accessible to all. Nat. Methods.

[21] Valeur A., Tunlid A., Odham G. (1988). Differences in lipid composition between free-living and initially adhered cells of a Gram-negative bacterium. Arch. Microbiol..

[22] Prabhune A., Fox S.R., Ratledge C. (2002). Transformation of arachidonic acid to 19-hydroxy- and 20-hydroxy-eicosatetraenoic acids using *Candida bombicola*. Biotechnol. Lett..

[23] Lounds C., Eagles J., Carter A.T., MacKenzie D.A., Archer D.B. (2007). Spore germination in *Mortierella alpina* is associated with a transient depletion of arachidonic acid and induction of fatty acid desaturase gene expression. Arch. Microbiol..

[24] Lindqvist Y., Huang W., Schneider G., Shanklin J. (1996). Crystal structure of delta9 stearoyl-acyl carrier protein desaturase from castor seed and its relationship to other di-iron proteins. EMBO J..

[25] Feng Y., Wang Y., Liu J., Liu Y., Cao X., Xue S. (2017). Structural insight into acyl-ACP thioesterase toward substrate specificity design. ACS Chem. Biol..

[26] Nagao K., Murakami A., Umeda M. (2019). Structure and function of Δ9-fatty acid desaturase. Chem. Pharm. Bull..

[27] Srikanta Dani K.G., Hatti K.S., Ravikumar P., Kush A. (2011). Structural and functional analyses of a saturated acyl ACP thioesterase, type B from immature seed tissue of *Jatropha curcas*. Plant Biol..

[28] Weber M.H.W., Klein W., Müller L., Niess Ulf M., Marahiel M.A. (2001). Role of the *Bacillus subtilis* fatty acid desaturase in membrane adaptation during cold shock. Mol. Microbiol..

[29] Cao Y., Yang J., Xian M., Xu X., Liu W. (2010). Increasing unsaturated fatty acid contents in *Escherichia coli* by coexpression of three different genes. Appl. Microbiol. Biotechnol..

[30] Aguilar P.S., de Mendoza D. (2006). Control of fatty acid desaturation: A mechanism conserved from bacteria to humans. Mol. Microbiol..

[31] Annous B.A., Kozempel M.F., Kurantz M.J. (1999). Changes in membrane fatty acid composition of *Pediococcus* sp. strain NRRL B-2354 in response to growth conditions and its effect on thermal resistance. Appl. Environ. Microbiol..

[32] Hazel J.R., Eugene Williams E. (1990). The role of alterations in membrane lipid composition in enabling physiological adaptation of organisms to their physical environment. Prog. Lipid Res..

[33] Leekumjorn S., Cho H.J., Wu Y., Wright N.T., Sum A.K., Chan C. (2009). The role of fatty acid unsaturation in minimizing biophysical changes on the structure and local effects of bilayer membranes. Biochim. Biophys. Acta-Biomembr..

[34] Ohlrogge J., Browse J. (1995). Lipid biosynthesis. Plant Cell.

[35] Kachroo P., Shanklin J., Shah J., Whittle E.J., Klessig D.F. (2001). A fatty acid desaturase modulates the activation of defense signaling pathways in plants. Proc. Natl. Acad. Sci. USA.

[36] Salas J.J., Ohlrogge J.B. (2002). Characterization of substrate specificity of plant FatA and FatB acyl-ACP thioesterases. Arch. Biochem. Biophys..

[37] De Carvalho C.C.C.R., Caramujo M.J. (2018). The various roles of fatty acids. Molecules.

